# Urinary Dimethylamine (DMA) and Its Precursor Asymmetric Dimethylarginine (ADMA) in Clinical Medicine, in the Context of Nitric Oxide (NO) and Beyond

**DOI:** 10.3390/jcm9061843

**Published:** 2020-06-12

**Authors:** Dimitrios Tsikas

**Affiliations:** Core Unit Proteomics, Institute of Toxicology, Hannover Medical School, 30623 Hannover, Germany; tsikas.dimitros@mh-hannover.de; Tel.: +49-511-532-3984

**Keywords:** asymmetric dimethylarginine, dimethylamine, disease, fish, seafood

## Abstract

Asymmetric protein-arginine dimethylation is a major post-translational modification (PTM) catalyzed by protein-arginine methyltransferase (PRMT). Regular proteolysis releases asymmetric dimethylarginine (ADMA). Of the daily produced ADMA, about 10% are excreted unchanged in the urine. The remaining 90% are hydrolyzed by dimethylarginine dimethylaminohydrolase (DDAH) to L-citrulline and dimethylamine (DMA), which is readily excreted in the urine. The PRMT/DDAH pathway is almost the exclusive origin of urinary ADMA and the major source of urinary DMA. Dietary fish and seafood represent additional abundant sources of urinary DMA. The present article provides an overview of urinary ADMA and DMA reported thus far in epidemiological, clinical and pharmacological studies, in connection with the L-arginine/nitric oxide (NO) pathway and beyond, in neonates, children and adolescents, young and elderly subjects, males and females. Discussed diseases mainly include those relating to the renal and cardiovascular systems such as peripheral arterial occlusive disease, coronary artery disease, chronic kidney disease, rheumatoid arthritis, Becker muscular disease, Duchenne muscular disease (DMD), attention deficit hyperactivity disorder (ADHD), and type I diabetes. Under standardized conditions involving the abstinence of DMA-rich fresh and canned fish and seafood, urinary DMA and ADMA are useful as measures of whole-body asymmetric arginine-dimethylation in health and disease. The creatinine-corrected excretion rates of DMA range from 10 to 80 µmol/mmol in adults and up to 400 µmol/mmol in children and adolescents. The creatinine-corrected excretion rates of ADMA are on average 10 times lower. In general, diseases are associated with higher urinary DMA and ADMA excretion rates, and pharmacological treatment, such as with steroids and creatine (in DMD), decreases their excretion rates, which may be accompanied by a decreased urinary excretion of nitrate, the major metabolite of NO. In healthy subjects and in rheumatoid arthritis patients, the urinary excretion rate of DMA correlates positively with the excretion rate of dihydroxyphenylglycol (DHPG), the major urinary catecholamines metabolite, suggesting a potential interplay in the PRMT/DDAH/NO pathway.

## 1. Introduction-A Brief Historical Retrospect and Aim of the Review

**Figure 1:**
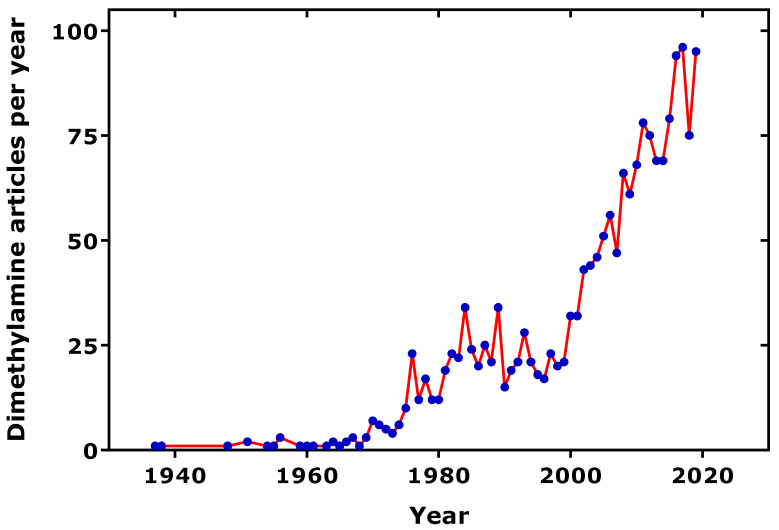
Number of dimethylamine-related articles published per year, as found in the PubMed (ncbi.nlm.nih), using the term “dimethylamine”. One average, 10% of the articles are related to urine.

Dimethylamine (DMA) is a natural compound which is widely distributed in the animal kingdom. The occurrence of DMA in human urine has been reported in 1935 and 1938 [1,2]. Subsequently, DMA was identified and measured in several biological fluids of humans and animals, especially including marine animals, suggesting that the part of DMA in human biological fluids may also originate from food. The scientific interest in DMA is still growing (Figure 1), not least because of its early recognized potential to form cancerogenic *N-*nitro(so)amines [3,4], notably in association with dietary nitrite. Due to the widespread interest in DMA, several authors have reviewed the importance of DMA for human health and disease from several perspectives. The focus of the present article is on the significance of urinary DMA from a clinical medicine point of view, beyond other important health issues such as cancer [5]. This review considered articles published in PubMed (ncbi.nlm.nih) relating to urinary DMA and its precursors from endogenous metabolism, including post-translational modification, i.e., asymmetric protein-arginine dimethylation, and food. Special emphasis was given to clinical studies on neonates, children and adolescents, adults reporting on urinary DMA, its precursor asymmetric dimethylarginine (ADMA), and related L-arginine metabolites in health and various diseases of the renal and cardiovascular systems. Clinical-pharmacological studies targeting key enzymes involved in the biosynthesis of ADMA and its metabolism to DMA were also considered.

## 2. Post-translational Modification and Food: Two Major Origins of Urinary DMA

**Figure 2:**
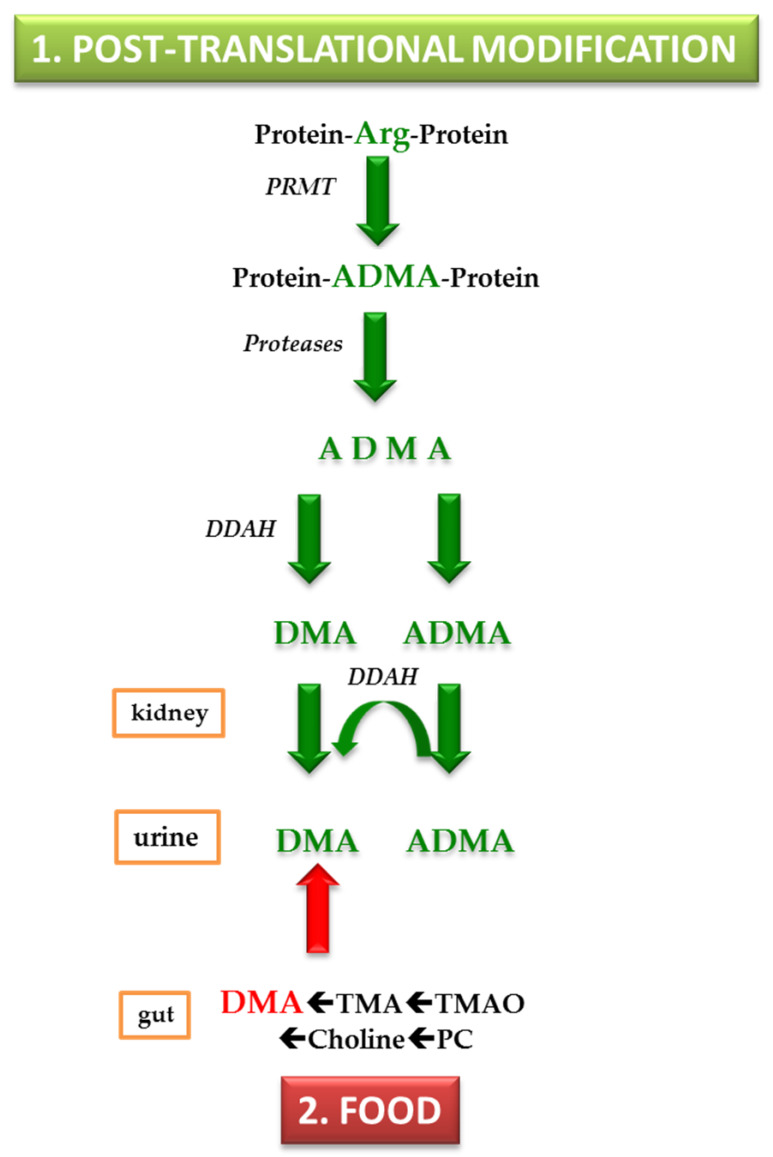
Simplified schematic of two major origins contributing to urinary dimethylamine. Abbreviations: PRMT, protein-arginine *N*^G^-methyl transferase; Arg, L-arginine; ADMA, asymmetric dimethylarginine; DDAH, dimethylarginine dimethylaminohydrolase. DMA, dimethylamine; TMA, trimethylamine; TMAO, trimethylamine *N*-oxide; PC, phosphatidyl choline.

Today, two major origins of circulating and urinary DMA in humans are established and will be discussed in this work. These are: (1) the cellular post-translational modification (PTM) of L-arginine (Arg) moieties in certain proteins; and (2) food, notably fish and seafood, which contain considerable amounts of phosphatidyl choline (PC), choline, trimethylamine *N*-oxide (TMAO) and trimethylamine (TMA), all being precursors of urinary DMA (Figure 2).

### 2.1. Asymmetric Protein-Arginine Dimethylation as the Major Contributor to Urinary DMA

Arg residues in proteins are methylated on their guanidine (*N*^G^) group by protein-arginine *N*^G^-methyltransferase (PRMT) (Figure 2). Their proteolysis releases *N*^G^-monomethylarginine (MMA), asymmetric dimethylarginine (*N*^G^,*N*^G^-dimethylarginine, ADMA) and symmetric dimethylarginine (*N*^G^,*N*′^G^-dimethylarginine, SDMA) (for recent reviews see [6,7]). ADMA has received particular attention, because it is an endogenous inhibitor of nitric oxide synthase (NOS) activity [8,9]. NOS converts Arg to nitric oxide (NO) and L-citrulline [8,9]. NO is one of the most potent endogenous vasodilators and regulators of blood pressure [10]. In adults, elevated circulating ADMA concentrations are associated with hypertension [10]. High plasma and low urinary ADMA concentrations are considered cardiovascular risk factors and have emerged as predictors of cardiovascular events and death in a range of diseases in adults [11,12]. The ADMA-related cardiovascular risk is generally assumed to be deeply rooted in the ability of ADMA to inhibit the NOS-catalyzed synthesis of NO in the endothelium [8,9,10,11,12]. Diminished NO bioavailability resulting from the inhibition of endothelial NOS activity by elevated ADMA concentrations may lead to hypertension in adults. Yet, the cardiovascular risk of ADMA is hypothesized to include other factors that are not yet known [8].

In humans, MMA and ADMA are hydrolyzed by dimethylarginine dimethylaminohydrolase (DDAH) to form monomethylamine and DMA, respectively (Figure 2). SDMA is not metabolized by DDAH. This enzyme was first discovered in rats, which were found to metabolize ADMA to DMA [13,14]. In adults, about 90% of daily produced ADMA are estimated to be hydrolyzed to DMA, with the remaining 10% being excreted unchanged in the urine (Figure 2). DMA circulates in blood in the lower µM-range and is excreted in the urine in the upper µM-range. Radiolabeled DMA (^14^C-DMA), administered orally to male volunteers, was found to be excreted in the urine by 87% during the first 24 h and by 94% after 3 days. These observations suggest that ^14^C-DMA is extensively absorbed from the gastrointestine and is excreted in the urine without appreciable metabolism [15]. The average urinary excretion of DMA in 203 healthy subjects, who maintained a normal diet, was measured to be 17.4 mg per day, corresponding to about 387 µmol DMA per day [16].

### 2.2. Food as Considerable Contributor to Urinary DMA

Fish and seafood are considerable contributors to urinary DMA. DMA excretion in the urine may increase up to 7-fold after the ingestion of fish and seafood, with the contribution of fruits, vegetables, dairy and grain products being rather negligible [17] (Figure 2). A likely precursor of DMA is trimethylamine *N*-oxide (TMAO). In the rat, administered radiolabeled TMAO (^14^C-TMAO) was found to be excreted in part unchanged and in part as ^14^C-DMA [18]. In the same study, TMAO was found to be converted to DMA when incubated with the colon and rectum, indicating bacterial activity. TMAO was found in deep-sea teleost fishes and skates, with their TMAO content correlating with depth [19,20,21,22]. It is assumed that TMAO is a key osmolyte in marine animals, playing important physiological roles, including the counteraction effects of hydrostatic pressure [23,24]. The severalfold increase in DMA excretion in the urine after the ingestion of fish and seafood [17] could be due to their depth-dependent high TMAO content (70–288 mmol/kg).

The additional dietary origins of TMAO and DMA are egg lecithin, choline and carnitine (Figure 2). In humans, the ingestion of 10 g choline chloride was found to increase the 24-h excretion of DMA twofold (from 340 µmol to 776 µmol) [25], suggesting that less than 1% of choline is excreted in the urine as DMA. Oral administration of 100 mg choline chloride to rats increased the creatinine-corrected urinary excretion of DMA twofold (94.5 µM/mM–185 µM/mM) [25]. In contrast, the intra-peritoneal injection of 100 mg choline chloride in rats did not result in appreciable increase of the creatinine-corrected DMA excretion rate (from 90.4 to 96.2 µM/mM) [25]. In the same study, the ingestion of 1 g egg lecithin by three rats increased the creatinine-corrected DMA excretion from 44 ± 12 to 74 ± 36 µM/mM after one day and to 198 ± 44 µM/mM after several days, suggesting that this phospholipid may be another source of DMA. In that study, TMA was found to contribute to urinary DMA. Interestingly, rats treated with neomycin were found to excrete about 20% less DMA, suggesting that the majority of urinary DMA is derived from endogenous metabolism [25]. Essentially, these observations were confirmed in rats by others, who found considerable concentrations of DMA in rat blood (19 µM) and rat gastric juice (33 µM) [26]. The serum concentration of DMA was measured to be on average 3.3 µM in healthy subjects and 29.1 µM for dialysis patients [27].

## 3. Urinary DMA and ADMA in Children, Adolescents and Adults in Health and Disease

It results from the above that in humans, the DDAH-catalyzed hydrolysis of endogenous ADMA generated by the PTM of Arg residues in proteins contributes up to 90% to urinary DMA, with the remaining fraction originating from dietary precursors. The complete avoidance of dietary sources of DMA is obviously not possible. Yet, under standardized and controlled conditions, i.e., avoidance of fresh and canned fish and seafood, urinary DMA may be a useful measure of whole-body ADMA synthesis, and hence of asymmetric Arg dimethylation. In clinical studies from our group and others, the volunteers have been asked to abstain from fish consumption and food rich in nitrate, due to complementary measurements of circulating nitrite and nitrate as measures of endogenous NO synthesis for Arg.

**Figure 3:**
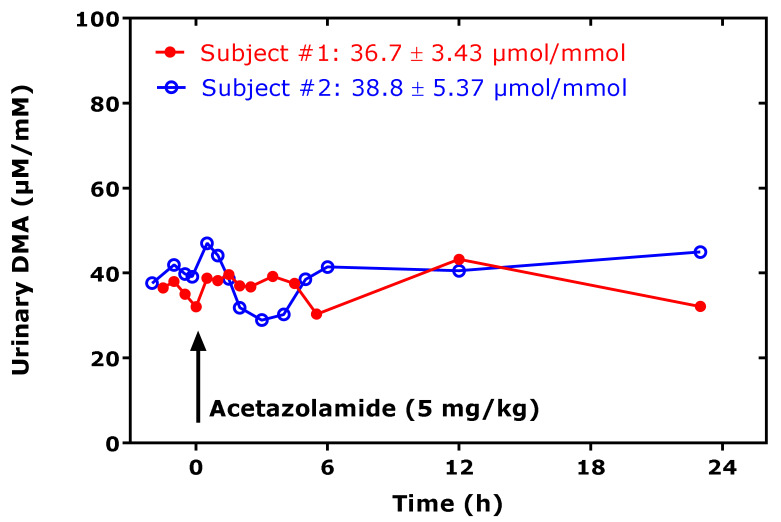
Creatinine-corrected excretion rate of dimethylamine (DMA) in urine spot urine samples collected by two healthy subjects at the indicate time points, before and after ingestion of an acetazolamide tablet (indicated by the arrow), at the dose of approximately 5 mg/kg bodyweight. The DMA excretion rate varied by 9% in subject # 1 and by 14% in subject # 2 over about 24 h.

In experimental and clinical studies, collection of urine for a considerable period of time, for example for 24 h, is not always feasible, notably in pediatric studies. Thus, another requirement for using urinary DMA as a measure of whole-body ADMA synthesis is the correction of the urinary concentration of DMA by the urinary concentration of creatinine measured in the same spot urine samples. The diurnal variation of DMA excretion in the urine is fairly constant, and drugs, such as the diuretic acetazolamide, which act in the proximal tubule of the nephron, do not affect the urinary excretion rate of DMA in healthy humans [28] (Figure 3).

Table 1 summarizes the urinary excretion rates of DMA and ADMA, as well as their approximate molar ratios in healthy and diseased children, adolescents and adults, as reported in the literature in chronological order, starting with the newest. Table 1 also lists the molar ratio of DMA to the sum of DMA and ADMA, i.e., DMA/(DMA + ADMA), as a surrogate for the relative whole-body DDAH activity.

In studies from our group, we used stable-isotope labeled gas chromatography-mass spectrometry (GC-MS)-based techniques for the measurement of urinary DMA [(CH_3_)_2_NH] [28], ADMA [29] and creatinine [30]. In our GC-MS method for urinary DMA, we use commercially available hexadeutero-DMA [(CD_3_)_2_NH] as the internal standard and their extractive derivatization with pentafluorobenzoyl chloride [28]. The analytical methods used by other groups are mentioned in places. The content of Table 1 is discussed below.

In 115 children and adolescents from Taiwan with chronic kidney disease (CKD) and a median age of 11.3 years, median creatinine-corrected urinary DMA excretion rates were measured to be 222 ng/mg creatinine in CKD stage G1 and 197 ng/mg in CKD stages G2–G4 [31]. On a molar basis, these values correspond to about 0.57 µmol/mmol and 0.51 µmol/mmol, respectively. These excretion rates belong to the lowest (about 60-fold lower) reported for DMA thus far (Table 1). In this cohort, creatinine-corrected TMAO excretion rates were determined to be 271 ng/mg and 184 ng/mg, corresponding to 0.41 µmol/mmol and 0.21 µmol/mmol, respectively. These excretion rates also belong to the lowest reported for TMAO in humans thus far. The families of the children and the adolescents were directed to have their children avoid of excessive intake of foods rich in choline and carnitine (e.g., eggs, fish, or red meat) for one week before blood and urine sampling. Yet, such a diet is unlikely to be responsible for the very low DMA and TMAO excretion rates [31]. The authors of this study concluded that TMA and DMA are superior biomarkers to TMAO, and that TMA and DMA are gut microbiota-dependent [31]. In our opinion, this conclusion does not hold true for DMA, at least not to a very large part, for the reasons discussed above. It is possible that DMA is more related to blood pressure abnormalities and cardiovascular risk in pediatric CKD as a metabolite of ADMA, rather than as a gut microbiota metabolite independent of ADMA. The same group has previously reported similarly low values in another childhood CKD cohort [32]. Yet, in previous study from the same authors’ group on 45 children and adolescents with CKD, much higher DMA and ADMA excretion rates were measured [33]. These considerable discrepancies are presumably due to the use of different analytical methods to measure urinary DMA, i.e., high-pressure liquid chromatography (HPLC) with fluorescence detection [33] versus liquid chromatography-tandem mass spectrometry (LC-MS/MS )[31] (Table 1).

In 23 children with attention deficit hyperactivity disorder (ADHD), we measured creatinine-corrected urinary excretion rates of 45.7 µmol/mmol for DMA and 6.2 µmol/mmol for ADMA, corresponding to an approximate DMA/ADMA molar ratio of 7.4 [34] (Table 1). In 19 ADHD children who were treated with methylphenidate, the corresponding values were 43.3, 5.6 and 7.7, with no statistical difference between treated and untreated children [34]. Comparable excretion rates and ratios were observed in black and white children, in black and white young men, and in black and white women (Table 1), indicating minimal ethnic differences with respect to DMA and ADMA [35].

In 11 healthy overweight men, the measured creatinine-corrected urinary excretion rates were 26.9 µmol/mmol for DMA and 3.59 µmol/mmol for ADMA, and the DMA/ADMA molar ratio was 7.49 at baseline (Table 1). The ingestion of three high-fat protein meals did not change these values, suggesting no postprandial effects on whole-body asymmetric protein-arginine dimethylation [36]. In that study, the concentration of DMA correlated strongly after Spearman (*r*_S_ = 0.883, *p* < 0.0001), with the concentration of creatinine in the urine samples [36].

In patients with Becker muscular dystrophy (BMD) the measured baseline creatinine-corrected urinary excretion rates were 48.8 (group I) and 53.0 (group II) µmol/mmol for DMA and 5.75 (group I) and 8.05 (group II) µmol/mmol for ADMA, and the corresponding DMA/ADMA molar ratios were 8.49 and 6.58 (Table 1) [37]. Treatment of the BMD patients with metformin, L-citrulline and their combination did not result in appreciable changes in the urinary excretion rates of DMA and ADMA or their ratio [37].

**Figure 4:**
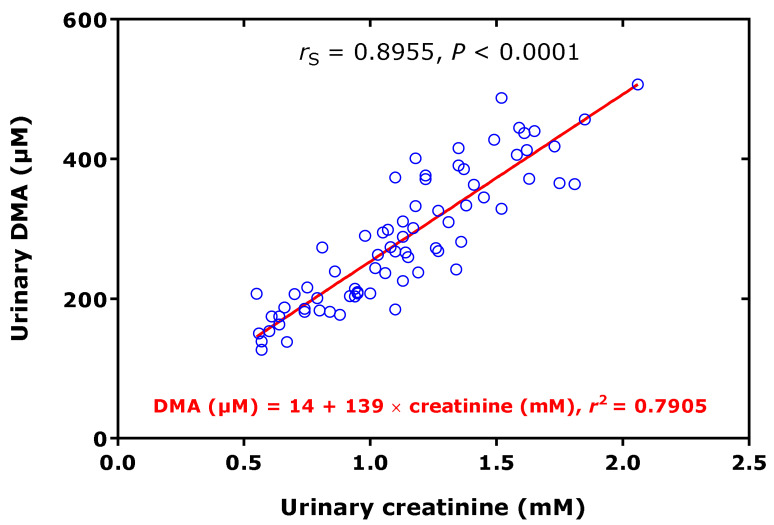
Spearman correlation (*r*_S_) and linear regression analysis between the dimethylamine (DMA) and creatinine concentrations measured in urine samples of 73 preterm neonates [38].

**Figure 5:**
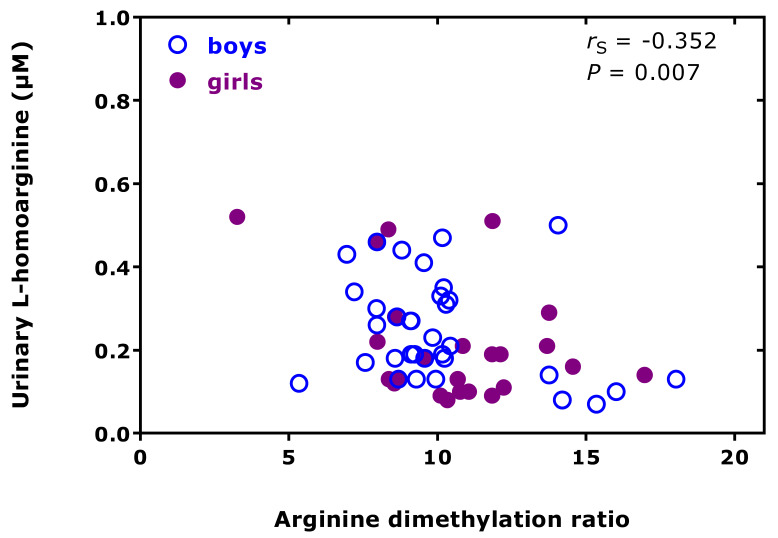
Spearman correlation (*r*_S_) between the urinary excretion of L-homoarginine and the arginine dimethylation ratio, the molar ratio of the sum of the urinary concentration of asymmetric dimethylarginine (ADMA) and dimethylamine (DMA) and of the urinary concentration of symmetric dimethylarginine (SDMA), i.e., (ADMA+DMA)/SDMA, in preterm neonates [38].

In preterm neonates, high creatinine-corrected excretion rates were measured for DMA (256 µmol/mmol) and ADMA (12.8 µmol/mmol), with an average DMA/ADMA molar ratio of 20.2 (Table 1) [38]. These values belong to the highest measured by our group using the same GC-MS-based approaches. In this cohort, we found a high correlation between the concentrations of DMA and creatinine measured in the urine samples (Figure 4). In the preterm neonates, the ratio of the whole-body asymmetric Arg dimethylation to the whole-body symmetric Arg dimethylation, measured as the molar ratio of the sum of urinary ADMA and urinary DMA (i.e., ADMA + DMA) and of SDMA, i.e., (ADMA + DMA)/SDMA, was found to be inversely associated with the urinary concentration of L-homoarginine (hArg) in the whole cohort (Figure 5), but not in the boys (*r*_S_ = −0.233, *p* = 0.184) or in the girls (*r*_S_ = −0.188, *p* = 0.379).

High correlations between DMA and creatinine concentrations in urine were also observed in black and white boys (*r*_S_ = 0.868, *p* < 0.0001; *n* = 79), as well as in black and white young men and women (*r*_S_ = 0.925, *p* < 0.0001, *n* = 1025). The difference between black (*n* = 41) and white boys (*n* = 39) with respect to the relative DDAH activity were very small (1.4%), but statistically significant (0.861 vs 0.873, *p* = 0.012).

In elderly patients suffering from peripheral arterial occlusive disease (PAOD) or coronary artery disease (CAD), we measured high DMA and ADMA excretion rates. The DMA/ADMA molar ratio was about 2 times higher in PAOD compared to CAD (Table 1) [39].

In several pediatric studies, we measured biomarkers of the L-arginine/NO pathway, including urinary DMA and ADMA.

In 55 children with Duchenne muscular dystrophy (DMD), we measured high creatinine-corrected DMA (78.9 µmol/mmol) and ADMA (20.3 µmol/mmol) excretion rates, which resulted in the very low DMA/ADMA molar ratio of 3.9 (Table 1) [40]. The urinary excretion of DMA (*r*_S_ = 0.522, *p* < 0.0001) and ADMA (*r*_S_ = 0.652, *p* < 0.0001) correlated positively with the DMD disease stage (after Vignos and Thompson). In that study, steroid medication decreased the plasma ADMA concentration (by 12%) as well as the ADMA (by 44%) and DMA (by 36%) urinary excretion rates. Creatine supplementation also decreased the DMA urinary excretion rate (by 29%). In both cases, the urinary nitrate excretion rate decreased upon treatment (by 38% and 34%, respectively) [40].

In 10 newly diagnosed (ND) children with type 1 diabetes mellitus (T1DM), we also found high creatinine-corrected excretion rates of DMA (40.3 µmol/mmol) and ADMA (10.2 µmol/mmol) and a very low DMA/ADMA molar ratio of about 4 (Table 1) [41]. In 92 T1DM children, the creatinine-corrected excretion rates were 30.5 µmol/mmol for DMA and 5.32 µmol/mmol for ADMA, and a higher DMA/ADMA molar ratio of about 6 (Table 1) [41]. The differences between newly diagnosed (i.e., untreated) and medicated T1DM children were significant for urinary ADMA (*p* = 0.003) and the DMA/ADMA molar ratio (*p* = 0.013), but not for DMA (*p* = 0.100) [41].

In a small group of children with haemolytic-uraemic syndrome (HUS), the creatinine-corrected excretion rates were 13.7 µmol/mmol for DMA and 3.3 µmol/mmol for ADMA, corresponding to a low DMA/ADMA molar ratio of about 4 (Table 1) [42]. In pediatric homocystinuria, high creatinine-corrected excretion rates of DMA (62.2 µmol/mmol) and ADMA (8.1 µmol/mmol) were measured, resulting in a rather “normal” DMA/ADMA molar ratio of 7.7 (Table 1) [43]. In 52 children with phenylketonuria (PKU), we only determined the ADMA excretion rate, which was comparable to those measured in other pediatric studies (Table 1) [43]. Compared to children with normocholesterolemia (normoCh, *n* = 54), children with hypercholesterolemia (hyperCh) type II (*n* = 64) were found to have higher creatinine-corrected DMA excretion rates and a higher DMA/ADMA molar ratio (each *p* = 0.0004) (Table 1) [44].

In 77 patients with CAD, we found comparable creatinine-corrected DMA excretion rates, independent of the number of vessel diseases (Table 1) [45]. The lowest creatinine-corrected excretion rate of ADMA and the highest DMA/ADMA molar ratio were observed in the CAD patients with 3-vessel disease (Table 1) [45]. This study revealed low urinary ADMA excretion rates as a predictor of mortality risk in patients with CAD [45].

In patients with rheumatic diseases, considerable differences were found for the creatinine-corrected excretion rates of DMA and ADMA and their molar ratio, both with respect to healthy subjects and within the individual variants of rheumatoid arthritis (RA), i.e., undifferentiated RA, spondyloarthritis and vasculitis (Table 1) [46]. The newly calculated relative DDAH activity in the patients of this study did not reveal any correlation with the urinary biomarkers of nitrosative and oxidative stress, 3-nitrotyrosine (*r* = 0.19, *p* = 0.32) and 8-iso-prostaglandin F_2α_ (8-iso-PGF_2α_) (*r* = −0.07, *p* = 0.73), respectively.

Liver and kidney are major eliminating organs for ADMA. In nine patients with end-stage liver disease, the mean creatinine-corrected DMA and ADMA excretion rates were 47.8 and 5.7 µmol/mmol, respectively, and the DMA/ADMA molar ratio was determined to be 8.4 (Table 1) [47]. During orthotopic liver transplantation, urinary ADMA excretion did not change, whereas the creatine-corrected DMA excretion rate and the DMA/ADMA molar ratio increased post-operatively [47].

In 9 children (5–18 years of age) with sporadic focal segmental glomerulosclerosis (FSGS), the mean creatinine-corrected excretion rates were measured to be 345 µmol/mmol for DMA and 41 µmol/mmol for ADMA, resulting in a mean DMA/ADMA molar ratio of 8.4 (Table 1) [48]. In 11 children with other renal diseases, the mean creatinine-corrected excretion rates were measured to be 130 µmol/mmol for DMA and 5.7 µmol/mmol for ADMA, resulting in a mean DMA/ADMA molar ratio of 22.8 (Table 1) [48]. In the sporadic FSGS non-haemodialyzed children, the plasma ADMA concentration was found to correlate inversely with the glomerular filtration rate (GFR; *r* = −0.784, *p* = 0.012); no such correlation was found in the non-FSGS children [48].

In eight children (age, three days to three years) with citrullinemia (range, 299–2092 µM), the mean creatinine-corrected excretion rates were measured to be 285 µmol/mmol for DMA and 26 µmol/mmol for ADMA, resulting in a mean DMA/ADMA molar ratio of 11 and were not different from those in age-matched healthy children (Table 1) [49].

In 10 patients (age, 3–30 years) with Schimke-immuno-osseous dysplasia (SIOD), the mean creatinine-corrected excretion rate of ADMA was measured to be 13.3 µmol/mmol and did not differ from that of 10 healthy age-matched controls (Table 1) [50]. Within the SIOD patients, the highest creatinine-corrected excretion rates of ADMA had SIOD patients without dialysis or renal transplantation, whereas renal transplanted patients had the lowest ADMA excretion rates [50].

In some studies, the urinary excretion of DMA in 24-h collected urine samples in healthy humans was measured (Table 1). Intravenous administration of ADMA to healthy males at a dose of 3 mg/kg, corresponding to about 210 mg ADMA and equivalent to 1000 µmol ADMA, resulted in an increase of urinary DMA, from 260 µmol per day to 560 µmol per day [51]. In other studies, the urinary excretion rate of DMA was in the same range [16,25]. Considering mean creatinine excretion rates of 12–20 mg in women and men, the reported creatinine-corrected excretion rates of DMA in those studies [25,27,51] is calculated to be 13 and 25 µmol DMA per mmol creatinine, i.e., within the ranges reported in Table 1.

**Figure 6:**
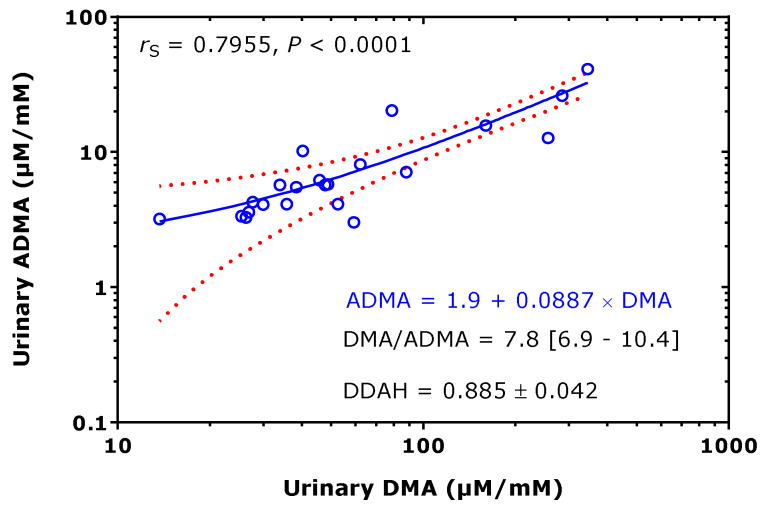
Spearman correlation and linear regression analysis between the creatinine-corrected excretion rates of asymmetric dimethylarginine (ADMA) and dimethylamine (DMA) of the data (22 pairs), taken from Table 1. Note the logarithmic scale on both axes. *r*_S_, Spearman coefficient of correlation. Doted curves indicate the 95th confidence interval. Dimethylarginine dimethylaminohydrolase (DDAH) = [DMA]/([DMA] + [ADMA]), relative whole-body DDAH activity.

**Figure 7:**
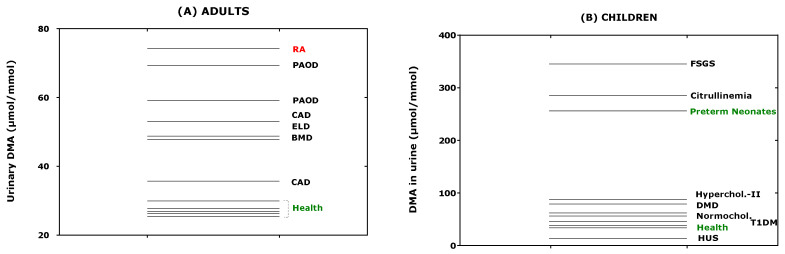
Creatinine-corrected excretion rates of dimethylamine (DMA) of the data taken from Table 1 for (**A**) adults and (**B**) neonates, children and adolescents. RA, rheumatoid arthritis; PAOD, peripheral arterial occlusion disease; CAD, coronary artery disease; ELD, end-stage liver disease; BMD, Becker muscular disease; FSGS, focal segmental glomerulosclerosis; DMD, Duchenne muscular disease; HUS, haemolytic-uraemic syndrome; T1DM, type 1 diabetes mellitus.

Considering the data of Table 1 available both for ADMA and DMA, their relationship was investigated. The creatinine corrected excretion rates of ADMA and DMA correlated strongly with each other after Spearman (*r*_S_ = 0.796) and the DMA/ADMA molar ratio was 7.8 (6.9–10.4) (median [IQR]; Figure 6). These observations support the close relation of DMA with ADMA in health and disease, in childhood and adulthood. The whole-body DDAH activity can be defined as the ratio of the excretion rate of DMA ([DMA]) and of the sum of the excretion rates of [DMA] and ADMA ([ADMA]). By using the data of Table 1, the mean whole-body DDAH activity is estimated to be 0.89 and to vary by about 5%. In preterm neonates [38], the whole-body DDAH activity was determined to be 0.955 ± 0.015 in the boys (*n* = 40) and 0.954 ± 0.012 in the girls (*n* = 34), and to be independent of gender (*p* = 0.698). To illustrate the considerable differences regarding the urinary DMA excretion rate between children/adolescents and adults, one the one hand, and health and disease, on the other side, the data listed in Table 1 are plotted in Figure 7, separately for adults and children.

Circulating and urinary ADMA and SDMA are considered cardiovascular risk factors in adults. Such evidence is still lacking in children and adolescents. An interesting study investigated whether young adult subjects who were born preterm with an extremely low birth weight differ in the ADMA catabolism compared to healthy young adults born at term [52]. The molar ratio of circulating ADMA and SDMA, i.e., ADMA/SDMA, was used in that study as a measure of ADMA catabolism. The ADMA/SDMA molar ratio was found to be higher in the ex-preterm-born adults (1.42 ± 0.31 vs. 0.95 ± 0.14, *p* < 0.0002). In the ex-preterm-born adults, the ADMA/SDMA molar ratio correlated inversely with birth weight (*r* = −0.68, *p* < 0.0001) and gestational age (*r* = −0.56, *p* < 0.0005) [52]. The authors of the study concluded that ADMA catabolism is significantly decreased in ex-preterm-born subjects. An alternative explanation for this finding could be a shift of the asymmetric-to-symmetric Arg-dimethylation in favor of SDMA [35].

## 4. Statins, Metformin, L-Arginine, Catecholamines and the PRMT-DDAH-NOS Axis inAtherosclerosis and Beyond

### 4.1. Effect of Statins on ADMA and NO Biosynthesis

Statins are pleiotropic drugs. They are powerful hepatic HMG-CoA (3-hydroxy-3-methyl-glutaryl-coenzyme A) reductase inhibitors and the most common cholesterol-lowering drugs. Statins are considered to reduce illness and mortality in patients at high risk of cardiovascular disease. In patients with hypercholesterolemia, rosuvastatin has been reported to lower the plasma concentration of ADMA [53], demonstrating, for the first time, that the endogenous synthesis of ADMA can be inhibited pharmacologically in vivo in humans. ADMA is an inhibitor of mobilization, differentiation, and function of endothelial progenitor cells (EPC); rosuvastatin was able to abolish the detrimental effects of ADMA [54]. In a rat model of isoproterenol-induced chronic heart failure, low-dose rosuvastatin was found to exert cardioprotective effects [55]. Isoproterenol (5 mg/kg) drastically increased the ADMA plasma concentration from 653 µM to 1030 µM and decreased NO synthesis in the heart, while rosuvastatin (5 mg/kg) reversed these effects and decreased the plasma ADMA concentration to 701 µM [55]. These observations were accompanied with an increase in ventricular PRMT1 expression and paralleled decreases in the ventricular expression of DDAH2 and eNOS [55]. Thus, isoproterenol and rosuvastatin are likely to be involved in, and to act oppositely in, the PRMT-DDAH-eNOS axis. A preliminary study in a rat model of isoproterenol-induced takotsubo cardiomyopathy (TTC) indicated that L-homoarginine administration may decrease the ADMA content in the heart tissue, possibly suggesting a cardioprotective effect of L-homoarginine [56], which is considered to counteract the detrimental effects of ADMA in the cardiovascular system [57]. The pharmacological effects of rosuvastatin beyond its cholesterol-lowering activity have been reviewed [58]. Endogenous catecholamines are considered to play a major role in TTC. Both NO generation and its effect were found to be accentuated in TTC patients [59]. In these TTC patients, plasma ADMA concentrations were lower and the responsiveness to NO (provided as sodium nitroprusside) on platelet function was found to be substantially greater than in controls, both acutely and after 3 months. In that study, plasma concentrations of ADMA and N-terminal-prohormone brain natriuretic peptide (NT-proBNP) were found to correlate with each other (*r* = 0.5, *p* = 0.003). Unfortunately, in the studies discussed above, no data were reported on circulating or urinary DMA.

### 4.2. Effect of Metformin on ADMA Biosynthesis

Metformin (1,1-dimethylbiguanide) is another pleiotropic, widely used type 2 diabetes mellitus (T2DM) drug. Metformin decreases glucose production in the liver, by mechanisms that are not yet fully understood. Metformin is partially structurally similar to DMA and ADMA. The organic cation transporter (OCT) 2 is primarily expressed in the kidney and mediates the first step in the reabsorption of endogenous small organic cations and basic drugs, such as metformin. At physiological urinary concentrations, DMA (IC_50_, 590 µM), TMA (IC_50_, 53 µM) and creatinine (IC_50_, 6.8 mM) were found to inhibit the in vitro transport of metformin [60], suggesting a potential role of DMA in the renal reabsorption of organic cations. Yet, pharmacological metformin (3 × 500 mg/d) did not change the DMA excretion in patients with Becker muscular dystrophy (BMD), suggesting no mutual interaction of metformin and DMA on the renal OCT2 activity [37]. Pharmacological acetazolamide (5 mg/kg), which is a strong inhibitor of the activity of carbonic anhydrase (IC_50_, 12 nM for carbonic anhydrase II), seems to also not affect the excretion of DMA in the proximal tubule (Figure 3).

**Figure 8:**
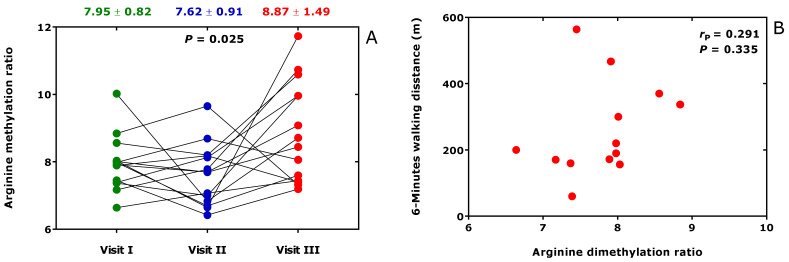
(**A**) Arginine dimethylation ratio, the molar ratio of the sum of the urinary concentration of asymmetric dimethylarginine (ADMA) and dimethylamine (DMA) and of the urinary concentration of symmetric dimethylarginine (SDMA) (ADMA + DMA/SDMA), at baseline (Visit I), after six-weeks treatment (Visit II) with metformin (3 × 500 mg/d) or L-citrulline (3 × 5000 mg/d) and subsequent six-weeks treatment (Visit II) with (3 × 500 mg/d) plus L-citrulline (3 × 5000 mg/d). (**B**) Relationship of the six-min walking distance in patients with Becker muscular dystrophy and the Arg dimethylation ratio at the end of the study (Visit III). This Figure was constructed with data reported elsewhere [37].

In BMD patients, the oral administration of metformin (3 × 500 mg/d) or L-citrulline (3 × 5000 mg/d) for six weeks (Visit II) followed of metformin (3 × 500 mg/d) plus L-citrulline (3 × 5000 mg/d) for another six weeks (Visit III) were found to exert, in part, different effects on the Arg/NO metabolism including the asymmetric and symmetric Arg dimethylation [37]. At the end of the study, the ratio of whole-body asymmetric Arg dimethylation to whole-body symmetric Arg dimethylation, measured as the molar ratio of the sum of urinary ADMA and urinary DMA (i.e., ADMA + DMA) and of SDMA (i.e., ADMA + DMA/SDMA), increased (Figure 8A). However, the 6-min walking distance was found not to correlate with the Arg dimethylation ratio ADMA + DMA/SDMA (Figure 8B).

### 4.3. Inflammation, Catecholamines and DMA Excretion

Generally, atherosclerosis is closely related to inflammation. In healthy overweight men, the basal urinary concentration of DMA correlated moderately with the plasma concentration of tumor necrosis factor alpha (TNFα) (*r*_S_ = 0.392, *p* = 0.029) and IL-6 (*r*_S_ = 0.370, *p* = 0.040), yet not with the plasma concentration of the cell adhesion molecules intercellular adhesion molecule-1 (ICAM-1), vascular adhesion molecule-1 (VCAM-1), E-selectin and P-selectin, suggesting a potential role of inflammation in DMA formation [36], i.e., with the activity of DDAH. In the African-PREDICT study, circulating IL-6 correlated positively with the excretion rate of DMA in the large cohort of healthy young black men, suggesting of a higher susceptibility of black people to inflammation.

**Figure 9:**
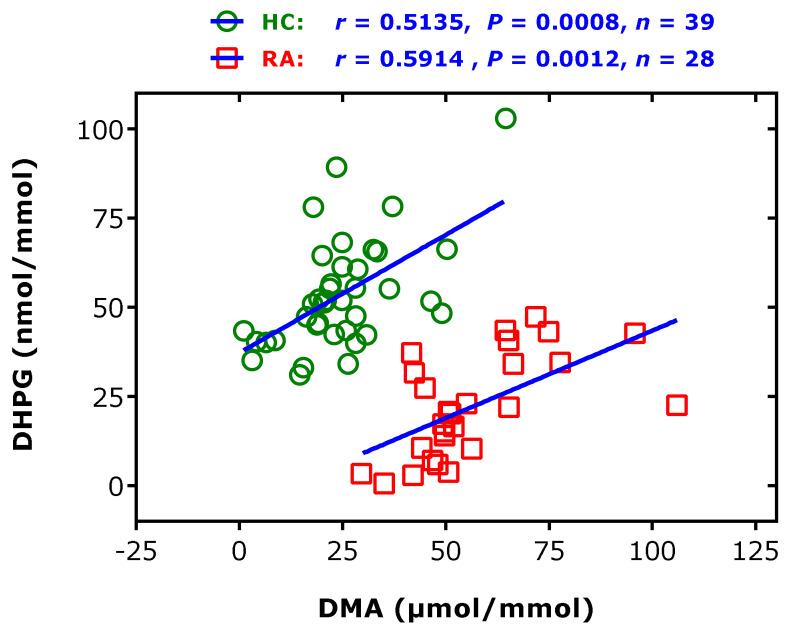
Spearman correlation and linear regression analyses between the creatinine-corrected excretion rates of dihydroxyphenylglycol (DHPG) and dimethylamine (DMA) in 39 healthy controls (HC) and in 28 subjects suffering from rheumatoid arthritis (RA). This Figure was constructed with data reported in part elsewhere [46,62].

Elevated creatinine-corrected DMA excretion rates were found in rheumatic diseases, notably in rheumatoid arthritis and spondyloarthritis [46] (Table 1). In patients with rheumatoid arthritis (*n* = 27), elevated creatinine-corrected excretion rates of DMA were found compared to healthy subjects (*n* = 39): 50.7 (45.0–65.3) vs 23.0 (18–28.8) µmol/mmol (*p* < 0.0001) [61]. In this cohort, the creatinine-corrected excretion rate of 3,4-dihydroxyphenylglycol (DHPG), the major urinary metabolite of the catecholamine norepinephrine (i.e., noradrenaline), was determined to be 20.7 (10.4–34.63) nmol/mmol in the patients and 51.4 (42.4–61.4) in the healthy subjects (*p* < 0.0001) [62]. These values indicate that the mean urinary excretion rate of DHPG is almost 1000 times lower than that of DMA. Interestingly, a correlation was observed between the excretion rates of DHPG, which is considered a biomarker of norepinephrine transporter inhibition [63,64], and DMA, in both study groups. Linear regression analysis between DHPG (*y*) and DMA (*x*) creatinine-corrected excretion rates resulted in the regression equations *y* = 37 + 0.662 × *x* (*r*^2^ = 0.3137, *p* = 0.0002) in the healthy subjects and *y* = −5.6 + 0.491 × *x* (*r*^2^ = 0.350, *p* = 0.0012) in the rheumatoid arthritis patients (Figure 9). These observations suggest a potential connection of the catecholamine and ADMA metabolism, with higher urinary DMA excretion indicating a higher catecholamine synthesis and vice versa (see also above). These findings are in line with observations in patients with end-stage kidney disease, showing a strong correlation between plasma ADMA and norepinephrine concentrations, and suggesting a common causal pathway, leading to cardiovascular disease in humans [65].

In conscious rats, ADMA and the synthetic NOS inhibitor *N*^G^-L-nitro-arginine-methyl ester (L-NAME) have been shown to activate the efferent sympathetic nerve activity, albeit at high infusion rates [66]. In patients with autonomic failure, NO synthesis was reported to be low in the supine position, causing hypertension, and high in supine positions, causing orthostatic hypotension [67]. The contribution of eNOS-derived NO to blood pressure in humans has been reported [68]. Lower plasma DHPG values have been reported in patients with orthostatic hypotension [69,70]. One may assume that ADMA does not activate the sympathetic nerve activity to the same extent in autonomic failure as in health. The relationship between the urinary excretion rates of DHPG and DMA in patients with autonomic failure is expected to be different than in healthy subjects and patients suffering from rheumatoid arthritis, but this remains to be investigated in this rare disease.

## 5. DDAH Activity and Oxidative Stress

**Figure 10:**
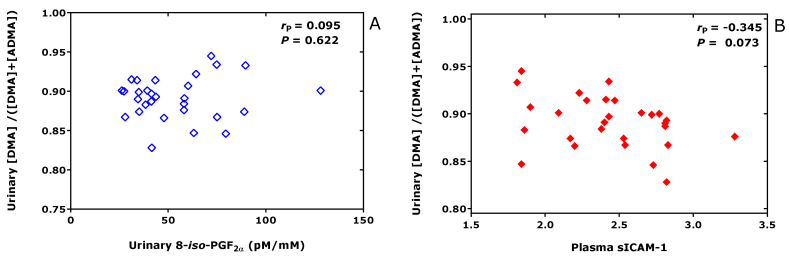
Pearson correlations between the whole-body DDAH activity, i.e., dimethylamine (DMA)/(DMA+ADMA), and the creatinine-corrected excretion rate or the oxidative stress biomarker 8-*iso*-prostaglandin F_2__α_ (8-*iso*-PGF_2__α_) (**A**) or plasma soluble intracellular adhesion molecule-1 (sICAM-1) (**B**) in healthy overweight men.

In vitro investigations suggested that a certain cysteine moiety in the catalytic center of DDAH is involved in the hydrolysis of ADMA to DMA and L-citrulline [71]. Generally, DDAH activity is believed to be sensitive to oxidative stress [72]. Yet, there is no solid evidence for this in humans in health and disease [73,74]. In healthy overweight men [36], we did not find any correlation between the whole-body DDAH activity (expressed as the molar ratio of urinary DMA to the sum of urinary DMA and ADMA) and the whole-body formation of the oxidative stress biomarker 8-*iso*-PGF_2__α_ [75] (expressed as creatinine-corrected excretion rate) (Figure 10A). In contrast, whole-body DDAH activity was found to be borderline inversely associated with the plasma concentration of soluble intracellular adhesion molecule-1 (sICAM-1) (Figure 10B), an inter-cellular adhesion molecule assumed to be a biomarker for inflammatory processes involving damage to platelets and endothelium and dyslipidemia [76].

## 6. DDAH as a Pharmacological Target

For about two decades now, DDAH has been the focus of pharmacologists aiming to modulate NO synthesis. In most diseases, notably of the renal and cardiovascular systems, elevated ADMA concentrations have been reported, which are thought to inhibit NOS activity. Thus, decreasing ADMA concentration by activating DDAH is an option to increase NO synthesis [77]. On the other hand, there are fewer diseases and conditions, such as sepsis, which may be associated with highly elevated NO synthesis. In such cases, the inhibition of DDAH activity by synthetic low-molecular-mass drugs has been considered as a pharmacological option. Pilot studies have demonstrated the principle utility of different pharmacological manipulations, including that targeting single nucleotide polymorphism of DDAH 2 [78]. Yet, given the potential of additional not yet fully recognized biological activities of free ADMA and ADMA-containing proteins [9], special attention must be given when targeting DDAH expression and/or activity. It should also be considered that the PRMT/DDAH/Arg/NO axis may also be linked to other pathways, including myeloperoxidase and the renin-angiotensin-aldosterone systems (RAAS), as observed in the cardiovascular risk patients of the LURIC study [79].

This review did not focus on asymmetric Arg-dimethylation of histone proteins. Recent experimental studies suggest that asymmetric Arg-dimethylation on histone H4 seems to be a senescence indicator and a potential anti-aging drug screening marker such as metformin [80], which also targets the H3K27me3 demethylase KDM6A/UTX [81].

## 7. Conclusions and Perspectives

Asymmetric and symmetric Arg-dimethylation is an abundant post-translational modification (PTM) catalyzed by PRMT. Few studies indicate that thus modified proteins have different biological activities than their native precursors. In addition, this PTM is a major source of free ADMA and SDMA, which emerged as cardiovascular risk factors, with the underlying mechanisms being still incompletely understood and may include inhibition of NO synthesis in the endothelium. Thus far, human erythrocytes were identified as a major source of ADMA-proteins. The physiological functions of ADMA-proteins in erythrocytes are unknown, but they are likely to contribute to their stability and fluidity. The understanding of the roles of Arg-dimethylation in erythrocytes and other cells in health and disease warrants further investigations.

A large fraction of ADMA released by proteolysis of ADMA-proteins is metabolized by DDAH to DMA, which is excreted in the urine. In particular, fish and seafood may also be considerable contributors to urinary DMA. Under dietary conditions minimizing their contribution to DMA, the measurement of DMA in urine is a useful measure of whole-body DDAH activity. The creatinine-corrected excretion rates of ADMA and DMA are higher in neonates and children than in adults, without evidence of endothelial dysfunction and the elevated inhibition of NO synthesis. There must be additional NO-independent effects of ADMA-proteins and free ADMA, presumably including the modulation of growth. This remains to be investigated in forthcoming studies.

ADMA and SDMA measurement in blood and urine is very popular and has helped identify their importance in pathophysiology. Yet, this information is incomplete, at least for ADMA, which is abundantly metabolized to DMA. Measurements of the urinary DMA and ADMA in humans indicate that the Arg/NO pathway and the asymmetric Arg-dimethylation are involved in many diseases. Unfortunately, in the context of the Arg/NO pathway and PTM, the DMA in urine is measured only by a very small number of investigator groups. Pharmacological studies aiming to modulate the PRMT/DDAH activity are also relatively rare and almost exclusively focused on the measurement of circulating ADMA. It is expected that the measurement of DMA in urine will provide valuable information, potentially leading to a more complete picture and understanding of the involved pathways. With respect to oxidative stress, there is no convincing evidence thus far that oxidative and nitrosative stress deteriorates DDAH activity in health and disease.

The intriguing correlation found between the excretion rates of DMA and DHPG in healthy subjects and in rheumatoid arthritis patients support a relationship between the PRMT/DDAH and catecholamine pathways. The underlying mechanisms warrant further investigations. Of particular interest in this context are rare diseases such as autonomic failure, in which the Arg/NO pathway and the catecholamines play major roles, for instance in regulating blood pressure.

## Figures and Tables

**Table 1:** Summary of reported creatinine-corrected urinary excretion rates of asymmetric dimethylarginine (ADMA) and dimethylamine (DMA) and of calculated DMA/ADMA molar ratio and the relative whole-body dimethylarginine dimethylaminohydrolase (DDAH) activity ((DMA/(DMA+ADMA)) in several conditions in humans.

Condition	DMA (µM/mM)	ADMA (µM/mM)	DMA/ADMA	Relative DDAH Activity	Ref.
Pediatric CKD (*n* = 115)	stage G1: 0.57 stage G2–4: 0.51	not measured	n.a.		[31]
Mild-to-Moderate Pediatric CKD (*n* = 55)	G1: 3.28 G2–3a: 2.47	G1: 16.9 G2–3a: 16.5	G1: 0.19 G2–3a: 0.15		[32]
Pediatric CKD (*n* = 45)	G1: 28 G2–4: 26.8	G1: 3.1 G2–4: 1.9	G1: 9.03 G2–4: 14.1		[33]
Pediatric ADHD (*n* = 23)	45.7	6.2	7.37	0.881	[34]
Black Children (*n* = 41) White Children (*n* = 39)	33.9 38.4	5.7 5.5	5.95 6.98	0.861 0.873	[35]
Black Young Men (*n* = 292) White Young Men (*n* = 281)	25.4 26.3	3.34 3.28	7.60 8.02	0.884 0.889	[35]
Black Young Women (*n* = 309) White Young Women (*n* = 312)	27.7 29.9	4.25 4.09	6.52 7.31	0.867 0.880	[35]
Healthy Overweight Men (*n* = 11)	26.9	3.59	7.49	0.882	[36]
BMD, Group I (*n* = 10) BMD Group II (*n* = 8)	48.8 53.0	5.75 8.05	8.49 6.58	0.895 0.953	[37]
Preterm Neonates (*n* = 73 or 75)	256	12.7	20.2	0.941	[38]
PAOD, Group I (*n* = 20) PAOD, Group II (*n* = 20)	59.2 69.3	3.02 2.89	19.6 24.0	0.951 0.897	[39]
CAD, Group I (*n* = 29) CAD, Group II (*n* = 31)	35.7 35.7	4.10 3.58	8.71 9.97	0.795 0.798	[39]
DMD (*n* = 55)	78.9	20.3	3.89		[40]
Pediatric T1DM ND (*n* = 10) Pediatric T1DM Treated (*n* = 92)	40.3 30.5	10.2 5.32	3.95 5.74		[41]
Pediatric HUS (*n* = 4 or 5)	13.7	3.3	4.15	0.811	[42]
Pediatric Homocystinuria (*n* = 6)	62.2	8.1	7.68	0.885	[43]
Pediatric PKU (*n* = 52)	not measured	6.8	n.a.		[43]
Pediatric HyperCh Type II (*n* = 64) Pediatric NormoCh (*n* = 54)	88 56	7.1 7.2	12.4 7.78	0.925	[44]
CAD (Stage 0/1/2/3) (*n* = 77)	52.5/53/53/53	4.1/4.3/4/3	13.7/13.3/15/22	0.928	[45]
Rheumatoid Arthritis RA (*n* = 10) Undifferentiated RA (*n* = 10) Spondyloarthritis (*n* = 5) Vasculitis (*n* = 3) Health (*n* = 10)	74.3 38.4 82.5 46.9 10.1	2.82 3.29 2.77 3.86 1.35	26.3 11.7 29.8 12.2 7.48		[46]
End-Stage Liver Disease (*n* = 9)	47.8	5.7	8.39	0.893	[47]
Pediatric FSGS (*n* = 9) Non-FSGS (*n* = 11)	345 130	41 5.7	8.41 22.8	0.894	[48]
Pediatric Citrullinemia (*n* = 8)	285	26.1	10.9	0.916	[49]
Schimke Disease (*n* = 10) Healthy Controls (*n* = 10)	not measured not measured	13.3 17.0	n.a. n.a.		[50]
Condition	**DMA** (µmol/d)	**ADMA**	**DMA/ADMA**		**Ref.**
Healthy Males (*n* = 6) Placebo ADMA Intravenous ADMA (3 mg/kg)	260 560	not measured not measured	n.a. n.a.		[51]
Healthy Females (*n* = 101) Healthy Males (*n* = 102) Healthy Humans	300 470 160–1280	not measured not measured not measured	n.a. n.a. n.a.		[16]
Healthy Men (*n* = 4)	340	not measured	n.a.		[25]

Abbreviations. CKD, chronic kidney disease; ADHD, attention deficit hyperactivity disorder; BMD, Becker muscular dystrophy; PAOD, peripheral arterial occlusive disease; CAD, coronary artery disease; Duchenne muscular dystrophy (DMD); T1DM, typ 1 diabetes mellitus; ND, newly diagnosed; HUS, haemolytic-uraemic syndrome; PKU, phenylketonuria; normoCh, normocholesterolemia; HyperCh, hypercholesterolemia; RA, rheumatoid arthritis; FSGS, focal segmental glomerulosclerosis; n.a., not applicable.
